# Ethanol-Induced Changes in PKCε: From Cell to Behavior

**DOI:** 10.3389/fnins.2018.00244

**Published:** 2018-04-12

**Authors:** Rashidi M. Pakri Mohamed, Mohd H. Mokhtar, Ernie Yap, Athirah Hanim, Norhazlina Abdul Wahab, Farah H. F. Jaffar, Jaya Kumar

**Affiliations:** ^1^Department of Family Medicine, Universiti Kebangsaan Malaysia, Kuala Lumpur, Malaysia; ^2^Department of Physiology, Faculty of Medicine, Universiti Kebangsaan Malaysia, Kuala Lumpur, Malaysia; ^3^Department of Medicine, Universiti Kebangsaan Malaysia, Kuala Lumpur, Malaysia

**Keywords:** PKC, PKCε, ethanol, epsilon, alcohol, RACK

## Abstract

The long-term binge intake of ethanol causes neuroadaptive changes that lead to drinkers requiring higher amounts of ethanol to experience its effects. This neuroadaptation can be partly attributed to the modulation of numerous neurotransmitter receptors by the various protein kinases C (PKCs). PKCs are enzymes that control cellular activities by regulating other proteins via phosphorylation. Among the various isoforms of PKC, PKCε is the most implicated in ethanol-induced biochemical and behavioral changes. Ethanol exposure causes changes to PKCε expression and localization in various brain regions that mediate addiction-favoring plasticity. Ethanol works in conjunction with numerous upstream kinases and second messenger activators to affect cellular PKCε expression. Chauffeur proteins, such as receptors for activated C kinase (RACKs), cause the translocation of PKCε to aberrant sites and mediate ethanol-induced changes. In this article, we aim to review the following: the general structure and function of PKCε, ethanol-induced changes in PKCε expression, the regulation of ethanol-induced PKCε activities in DAG-dependent and DAG-independent environments, the mechanisms underlying PKCε-RACKε translocation in the presence of ethanol, and the existing literature on the role of PKCε in ethanol-induced neurobehavioral changes, with the goal of creating a working model upon which further research can build.

## Introduction

Protein kinases C (PKCs) are a family of protein kinase enzymes that regulate most cellular reactions by controlling the function of other proteins through the phosphorylation of hydroxyl groups of serine and threonine amino acid residues (Ohno and Nishizuka, 2002). Generally, PKCs are divided into three classes: atypical aPKCs (PKCζ and PKCλ), conventional cPKCs (PKCα, PKCβ, and PKCγ), and novel nPKCs (PKCδ and PKCε). PKCs show considerable variations in their downstream targets, central nervous system (CNS) distribution, second messenger activators, and affinities toward substrates (Tanaka and Nishizuka, 1994; Newton and Johnson, 1998).

PKCε is a phorbol ester/diacylglycerol (DAG)-sensitive and calcium-independent serine/threonine kinase. PKCε is abundantly present in regions of the brain that are implicated in drug addiction, such as the frontal cortex, striatum, nucleus accumbens (NAc), and hippocampus (Saito et al., 1993; Minami et al., 2000). PKCε is considered to mediate an ethanol-tolerant phenotype because of its interactions with receptors such as gamma aminobutyric acid (GABA_A_) (Poisbeau et al., 1999) and metabotropic glutamate receptor subtype 5 (mGlu5) (Olive et al., 2005; Kumar et al., 2017) in CNS. Null mutations of PKCε have been found to attenuate ethanol drinking behavior in rodents (Lesscher et al., 2009; Maiya et al., 2016), and prolonged ethanol exposure significantly increases PKCε expression (Messing et al., 1991; Coe et al., 1996; Kumar et al., 2016). Thus, there is an incentive for developing potent, selective, and brain-penetrant PKCε inhibitors. However, the first step toward this goal is to elucidate the ethanol-associated PKCε-mediated signaling pathways.

The biology of PKCε is complex, and a systematic approach is required for appreciating its pivotal function in ethanol addiction. In the present review, we shall first discuss the general structure and function of PKCε and its localization in brain regions related to addiction phenotypes. PKCε expression could be controlled at the transcriptional, translational, or post-translational levels. PKCε needs to achieve catalytic maturity through its complete phosphorylation. In this article, we present a broad overview of the roles of upstream kinases, phosphatases, and activators of PKCε and their interactions with ethanol. Considering that activated PKCε travels to distinct subcellular locations to exert its effects, we examine the current state of understanding of ethanol-induced intracellular translocation of PKCε.

## Form fits function: general structure of PKCε and functional relevance

PKCε consists of a regulatory domain and a catalytic domain joined together by a hinge region. PKCε consists of three conserved regions, C1, C3, and C4, and five variable regions, V1–V5. C1 is known for its regulatory function and C3 and C4 for their catalytic activities (Newton and Ron, 2007; Newton and Messing, 2010). Intramolecular and intermolecular interactions of PKCε are regulated by its phosphorylation at Thr-566 in the activation loop, Ser-729 in the C-terminal hydrophobic region, and Thr-710 at an autophosphorylation site (Akita, 2002). The binding of a pseudosubstrate to the substrate-binding cavity maintains PKCε in the inactive conformation (Newton, 2001). Pseudosubstrates are naturally occurring autoinhibitory domains within the N-terminal regulatory region of PKCε. They function to maintain PKCε in the inactive state until the arrival of an appropriate signal, which then relieves the pseudosubstrate from the regulatory region (Steinberg, 2008). Numerous second messengers, including phosphatidylinositiol 3,4,5-triphosphate, DAG, and fatty acids (Moriya et al., 1996; Graneß et al., 1998), can act on the C1 domain and cause PKCε recruitment at various subcellular locations. PKCε can bind to specific substrates and affect downstream signaling events (Newton, 2001) through interaction with specific scaffolding or anchoring proteins known as RACKs to mediate ethanol-induced neurobehavioral changes (Ron et al., 1999).

## Ethanol-induced changes in expression of PKCε

Ethanol could affect PKCε activities through the regulation of its expression. By increasing PKCε expression, a higher reserve pool of PKCε is available to be phosphorylated and activated.

## Transcription of PKCε

PKCε is encoded by *PRKCE*, which is localized in chromosome 2p21 in humans (Basta et al., 1992), 6q12 in rats, and 17E4 in mice. The human version consists of 32 exons, whereas the rodent version contains 17 exons. In an animal model of cocaine addiction, methylation of CpG dinucleotides in the *Blhb2*-*, Pparg*-*, E2f*-*, Egr1*-, and *Sp1*-binding sites in the *PRKCE* promoter region was reported following the chronic use of cocaine (Zhang et al., 2009). Methylation of these binding sites significantly decreased *PRKCE* mRNA levels (Zhang et al., 2009). One of the aforementioned transcription factors, *Sp1*, has been shown to be downregulated following chronic ethanol exposure (Rulten et al., 2006). However, findings regarding PKCε gene expression have been rather inconclusive, with no definitive changes in *PRKCE* mRNA levels reported after long-term ethanol exposure (Kaiser et al., 2014; Kumar et al., 2016).

## Ethanol-induced changes in phosphorylation of PKCε: the role of upstream kinases

Maturity of PKCε relies on a series of phosphorylation events that it has to undergo at Thr566 (activation loop), Thr710 (turn motif), and Ser729 (hydrophobic motif) (Newton, 2003; Parker and Murray-Rust, 2004). PDK-1 is known to catalyze the phosphorylation of PKCε at Thr566, which subsequently triggers autophosphorylations of PKCε at both the turn and hydrophobic motifs (TM and HM, respectively) (Newton, 2001). PDK-1 is activated by the upstream kinase PI3K (Parekh et al., 2000; Cenni et al., 2002), a family of signal-transducing enzymes that are directly activated by G-protein-coupled receptors and tyrosine kinase receptors (Leevers et al., 1999). PI3K activation triggers a series of cellular reactions that recruit numerous downstream kinases, such as PDK-1 and mTOR (Yang et al., 2008). PI3K along with glutamate receptors has been reported to regulate synaptic plasticity (Daw et al., 2002; Perkinton et al., 2002), highlighting the important role of this kinase in the development of sensitivity toward many addictive substances (Izzo et al., 2002; Corl et al., 2005). Along these lines, binge drinking has been shown to significantly upregulate the phosphorylation state of p85α (a PI3K-binding motif) in the nucleus accumbens (Cozzoli et al., 2009). In humans, genetic variations in PIK3R1 (the gene encoding the regulatory subunit of PI3K) are associated with risky ethanol drinking behavior in adolescents (Desrivières et al., 2008).

The mammalian target of rapamycin complex 2 (mTORC2) has been shown to facilitate the phosphorylation of PKCε at TM and HM. mTORC2 components, such as rapamycin-insensitive companion of mTOR (rictor) and SAPK-interacting protein 1 (Sin-1), are important for TM and HM phosphorylation of PKCε (Ikenoue et al., 2008). In C2C12 mouse myoblasts, ethanol exposure significantly increases the mRNA and protein expression of mTORC2 components, such as rictor and Sin-1, as well as their associations with mTOR, resulting in increased mTORC2 kinase activity (Hong-Brown et al., 2012). However, no empirical evidence showing a relationship between mTORC2 and PKCε phosphorylation in the human brain is yet available. mTORC1, another multi-protein complex of mTOR, has been associated with abuse of various classes of drugs (Puighermanal et al., 2009; Neasta et al., 2010; Bailey et al., 2012). As for ethanol, mTORC1 was shown to mediate ethanol-related memory reconsolidation (Barak et al., 2013) and neuroadaptations underlying binge drinking behaviors (Liu et al., 2017). However, no biochemical link between mTORC1 and PKCε has been reported to date.

## Ethanol-induced changes in subcellular localization of PKCε

As PKCε is a key regulator of various signal-transducing events, its demand to be present in several subcellular locations is met by translocation of the kinase by isozyme-specific chauffeur proteins. Aberrant translocation of the kinase could miscue the signaling outputs and hence be detrimental to cellular physiology.

## Chauffeur for PKCε

RACK is a 30–36-kDA protein that belongs to a class of anchoring proteins that mediate the localization of PKCs (Mochly-Rosen et al., 1991). RACK1 is the selective RACK that anchors to PKC beta II (βIIPKC) (Ron et al., 1994), whereas RACK2 or εRACK is the selective RACK for PKCε (Csukai et al., 1997). Both proteins consist of seven WD40-motif repeat structures (Coyle et al., 2009), which are thought to be involved in scaffolding function and protein–protein interactions (Adams et al., 2011). It is noteworthy that RACK is not a substrate of PKC, but its binding with PKC isoforms increases its substrate phosphorylation (Robles-Flores et al., 2002). Disturbance in this RACK-PKC isoform interaction results in destabilization of the PKC and disruption of the substrate phosphorylation (Mochly-Rosen et al., 1991). The expression of RACK appears to be tightly regulated, with εRACK expression increasing by 70% when PKCε is overexpressed by 10-fold (Pass et al., 2001).

PKCε binds to RACKε via its C2 domain (Csukai et al., 1997). The competition between the RACK protein and an autoinhibitory sequence within the PKC releases the autoinhibitory binding. This in turn exposes the substrate-binding site (Ron and Mochly-Rosen, 1995). The interaction of PKCε and RACKε is vital for translocation of the complex to the Golgi apparatus (GA), where PKCε gets phosphorylated at HM (Ser729) (Csukai et al., 1997). β′-COP, a subunit of a coatomer abundantly present in the GA or Golgi/endoplasmic reticular intermediate compartment, has been reported to bind to the C2 domain of PKCε in RACKε-β′-COP form (Budas, 2012; Caino et al., 2012). A recent study reported the PKCε-RACKε-β′-COP complex to travel to the GA via a small GTP-binding protein ADP-ribosylation factor (ARF-1)-mediated pathway in NIH3T3 cells in an unstimulated state. The same study also reported that phorbol ester-dependent transport of PKCε-RACKε to the membrane surface is coatomer independent (Peterson and Stamnes, 2013), suggesting that the role of the coatomer in PKCε transportation pertains to the Golgi alone.

## Ethanol-induced translocation of PKCε

Under normal physiological conditions, PKCε is found in the perinuclear area. PKCε activation by phorbol ester has been shown to cause translocation of the isozyme from the perinuclear area to the nucleus. Ethanol exposure (50 mM for 48 h and 25 mM for 4 days) triggered PKCε to translocate from the perinuclear area to the cytoplasm, remain there as long as ethanol was present, and stay for 48 h after ethanol exposure, whereupon PKCε relocated to the perinuclear region. *In vivo* experiments corroborated this translocation of PKCε to cytosol after the brief introduction (10 min following administration) of ethanol (2 g/kg, 20% v/v) (Kumar et al., 2006). Further, Yao et al. (2008) showed that ethanol induces translocation of PKCε by εRACK to the cytosol and found that pseudoεRACK (selective PKCε agonist) activates PKCε; however, it does not cause translocation of PKCε to the cytosol. The amount of εRACK in the cytosolic compartment also increased concomitantly with the amount of PKCε, suggesting that PKCε and εRACK moved together after being treated with ethanol. The cotranslocation of the complex requires PKCε to be activated before binding with εRACK. Ethanol exposure causes translocation of PKCε and εRACK to the cytoplasm from the nucleus/perinucleus and Golgi apparatus/perinucleus, respectively (Yao et al., 2008).

## Steering PKCε away from phosphatases?

Phosphatases also regulate the phosphorylation status of kinases and subsequently, its activities. Active PKCs are recruited to the membrane, which causes PKCs to adopt an open conformation prone to dephosphorylation and downregulation (Leontieva and Black, 2004). Phosphatases such as PH domain and leucine-rich repeat protein phosphatases (PHLPP) dephosphorylate PKCs at HM, which destabilizes the kinase, causing further dephosphorylation at AL and TM by PP2A-type phosphatases. Then, dephosphorylated PKCs are degraded (Gao et al., 2008). Increased dephosphorylation of PKCε at HM (Ser729) in 3T3 and 3T6 cell lines upon cell passage was reported. These findings suggest that cell passage induces changes in the localization of PKCε, making it prone to dephosphorylation by a Ser729 phosphatase (England et al., 2001). The plausibility of chronic ethanol-induced translocation of PKCε away from “PHLPP sites” offers an interesting hypothesis to test.

## PKCε in ethanol-induced neurobehavioral changes

Ethanol addiction is a progressive brain disorder that is characterized by a pathological pattern of ethanol use that progresses through initial, habitual, and compulsive stages. Early stages of ethanol addiction are defined by changes in initial sensitivity and the development of acute functional tolerance toward the effects of ethanol, which can lead to loss of the righting reflex (showing ethanol-induced sedation) and ataxia (the motor-impairing effects of ethanol). The absence of PKCε in knockout mice increases both the duration of loss of the righting reflex and the extent of ataxia (Hodge et al., 1999; Wallace et al., 2007). It stands to reason, therefore, that PKCε facilitates the development of acute functional tolerance to ethanol. Tolerance facilitates binge ethanol drinking as drinkers experience diminished symptoms of intoxication despite higher blood ethanol levels. Studies employing PKCε-null mutant mice reported these mice to consume significantly less ethanol (Hodge et al., 1999) and even to exhibit an increased aversion to ethanol (Newton and Messing, 2007). More recently, selective chemical genetic inhibition of PKCε catalytic activity has proven successful in decreasing ethanol consumption in mice (Maiya et al., 2016). Biochemical studies have revealed PKCε to modulate ethanol consumption behavior by decreasing inhibitory GABAergic neurotransmission through the phosphorylation of the GABA_A_ ⋎2 subunit at S327 (Qi et al., 2007) and phosphorylation of the N-ethylmaleimide sensitive factor at S460 and T461 (Chou et al., 2010). PKCε-null mice also showed decreased operant self-administration, with no escalation of dopamine at NAc, following brief exposure to ethanol (1 and 2 mg/kg, i.p.), suggesting a crucial role of PKCε in reinforcing the effects of ethanol (Olive et al., 2000).

In addition to GABA, PKCε has also been implicated in group-I mGlu subtype 1/5-associated signaling to mediate binge ethanol intake, as the mGlu5 antagonist 2-methyl-6-(phenylethynyl)pyridine (MPEP) was found to decrease ethanol consumption in mice via a PKCε-dependent mechanism (Olive et al., 2005). A brain region-specific approach revealed that metabotropic glutamate subtype receptor 1 or 5 (mGlu1/5)-PKCε signaling at NAc and central amygdala (CeA) is crucial for the manifestation of binge drinking (Cozzoli et al., 2016). Molecular studies have found PKCε to regulate the trafficking of mGlu5 at NAc via direct phosphorylation of the receptor (Ko et al., 2012; Schwendt and Olive, 2017). The kinase decreases mGlu5 surface expression by causing its internalization (Schwendt and Olive, 2017). In parallel to this finding, PKC was shown to phosphorylate mGlu5 at S901 in the C-terminus of the receptor, disrupting calmodulin (CaM) binding to mGlu5 (because CaM stabilizes the surface expression of mGlu5) and enhancing binding of the E3 ligase seven in abstentia homolog (Siah-1A) to the receptor, which decreases the surface levels of mGlu5 (Moriyoshi et al., 2004; Ko et al., 2012). Because of this degradation of mGlu5 (Moriyoshi et al., 2004; Ko et al., 2012), PKCε is thought to maintain an intracellular pool of mGlu5 (Schwendt and Olive, 2017). To date, the exact ethanol-induced PKCε consensus site at the mGlu5 C-terminal is not well defined. Ethanol enhances the PKC phosphorylation of mGlu5 at Ser 890 (Minami et al., 1998). PKCε has been shown to phosphorylate mGlu5 at Ser 839 in astrocytes (Bradley and Challiss, 2011). Recently, decreased mGlu5 availability was reported in the limbic system of abstinent ethanol-dependent patients (Leurquin-Sterk et al., 2016). Given the role of PKCε in the trafficking of rodent limbic mGlu5, the investigation of PKCε-mediated trafficking of mGlu5 should perhaps be extended to humans.

Recently, we reported a significant escalation in the protein expression of native and phosphorylated PKCε (S729) in the amygdala of rats during ethanol withdrawal (EW)-induced anxiety. Acute administration of ethanol (2.5 g/kg, 20% v/v) attenuated the abstinence-induced anxiety without affecting the expression of phosphorylated (S729) and total PKCε in the amygdala. We hypothesized that PKCε in the amygdala does not play a direct role in the manifestation of EW-induced anxiety (Kumar et al., 2016). In agreement with our findings, Olive et al. reported no changes in c-fos expression following EW-induced seizure in the amygdala of PKCε-null mice (Olive et al., 2000). Intriguingly, a growing body of literature has reported amygdala PKCε to play a central role in the modulation of ethanol consumption (Olive et al., 2000; Lesscher et al., 2009; Cozzoli et al., 2016). Thus, it stands to reason that, at least in the amygdala, ethanol-induced neuroadaptation may have modulated PKCε to act differently during the various stages of alcohol addiction.

## Role of dag in ethanol-PKCε mediated changes

The interaction of ethanol with G-protein-coupled receptors results in the generation of second messenger molecules, such as inositol triphosphate (IP3) and DAG (Yao et al., 2008). Consequently, PKCε is activated after DAG binds to the DAG-sensitive C1 domain of the kinase (Stahelin et al., 2005). However, prolonged exposure to activators (e.g., DAG/phorbol ester) could downregulate PKCε through dephosphorylation and proteolysis (Cameron et al., 2009). This provokes the question: could ethanol regulate PKCε activities via DAG-independent mechanisms? Recently, Cozzoli et al. (2016) showed that in the CeA, PKCε may operate via a mGlu1-dependent pathway without involving G_α*q*/11_-mediated stimulation of phospholipase C (PLC) (which generates DAG) in regulating binge ethanol intake. Hence, the authors went on to suggest that PKCε in certain brain regions mediates binge drinking behavior through a DAG-independent signaling pathway (Cozzoli et al., 2016). To date, studies investigating chronic ethanol exposure have reported decreased phosphatidylinositol 4,5-biphosphate (PIP2)/PLC activities (Katsura et al., 1994; Pandey, 1996) and increased total and phosphorylated PKCε expression (S729) (Kaiser et al., 2014; Kumar et al., 2016). As per these findings, a host of studies conducted in ethanol-free environments have suggested that a baseline DAG level is sufficient to support PKC activation despite the upregulation of PKC levels (Hu et al., 1987; Housey et al., 1988; Obeid et al., 1990).

**Figure 1:**
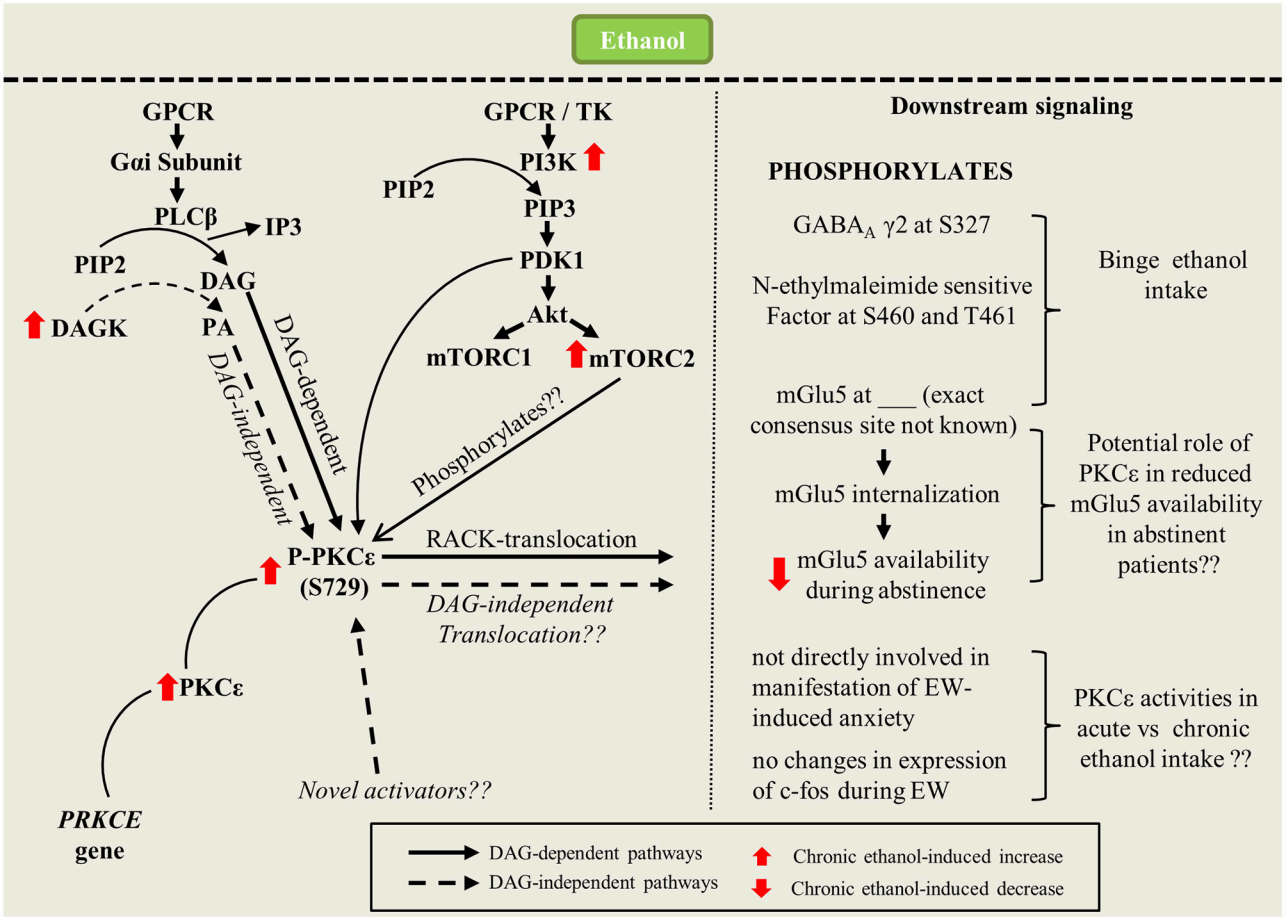
Signaling pathway of ethanol-mediated changes in PKCε activities (expression and translocation). Interaction of ethanol with GPCR results in generation of second messengers such as DAG and IP3. Long-term ethanol consumption upregulates cellular levels of basal PKCε and upstream kinases such as PI3K and mTORC2 which collectively increases phosohorylation of PKCε at S729. In DAG-dependent pathway, binding of DAG and RACK to mature PKCε translocates the kinase to distinct subcellular target sites to mediate downstream signaling pathways. In DAG-independent pathway, increase in DAG kinase activities causes generation of PA which could affect PKCε translocation via novel pathways. Once translocated, PKCε could affect downstream signaling through phosphorylation of numerous molecular targets which include GABA_A_, N-ethylmaleimide sensitif factor and mGlu5 which influence binge ethanol consumption. PKCε also causes internalization of mGlu5 surface receptors that could potentially reduce mGlu5 availability during abstinence.

Another vital research question is as follows: in a DAG-independent environment, how would PKCε be activated? Apart from being activated by DAG, PKCε could be activated by other compounds, such as phorbol esters or lipids like arachidonic acid and PtdInsP2 (Liu and Heckman, 1998; Shirai et al., 2007). The C2 domain of PKCε has been shown to bind to phospholipids, such as phosphatidic acid (PA), and this binding plays an important role in the membranal translocation of PKCε (Corbalan-Garcia et al., 2003; Jose Lopez-Andreo et al., 2003). PA is formed in several ways, one of which is through the phosphorylation of DAG by DAG kinase. Intriguingly, DAG kinase iota, which is found exclusively in the brain, was reported to be expressed 55% more in the cortex of alcohol-accepting rats than in non-alcohol-accepting rats (Sommer et al., 2001). Hence, the potential role of PA or other unknown compounds in PKCε activation should be explored further (Figure 1).

Findings related to DAG/phorbol ester affinities for the C1A and C1B domains of PKCε have also been somewhat inconsistent. Although some researchers have reported the affinity of phorbol ester to be three times higher for the C1B domain than for the C1A domain of PKCε (Stahelin et al., 2005), others have reported the affinity to be seven times higher (Irie et al., 1998, 2002). DiC18, a DAG analog, was shown to bind more readily to the C1A domain than to the C1B domain of the kinase (Stahelin et al., 2005). These inconsistencies have led to efforts directed toward discovering alternative effects of alcohol on the C1A and C1B domains of PKCε. In 2009, an allosteric alcohol-binding site in PKCε at the second cysteine-rich domain of C1B, which consists of His 236 and Tyr238, was identified (Das et al., 2009). More recently, multiple alcohol-binding sites on the C1A and C1B domains of PKCε were discovered (Pany and Das, 2015). These findings suggest an alternate mechanism for regulating PKCε activity by ethanol by the direct binding of ethanol to PKCε.

## Conclusion and future perspectives

Several intriguing insights have emerged from PKCε research: Ethanol-PKCε interactions might be DAG-free in some brain regions. Traditionally, ethanol has been thought to modulate PKCε activities either by increasing the production of lipid second messengers or by increasing the basal level of PKCε available to achieve catalytic maturity through alternate signaling pathways. The very notion that PKCε could operate in a DAG-independent environment suggests potential novel mechanisms in its activation and localization which should be further investigated. Except for the *Xenopus laevis* oocytes at Ser890, to date, we do not have knowledge of other ethanol-induced PKCε phosphorylation consensus sites on the C-terminal of mGlu5. Given the centrality of mGlu5 in modulating ethanol-induced neurobehavioral changes, future studies should be directed to identify the biochemistry and physiology of putative phosphorylation sites of PKCε at the mGlu5 C-terminal. The existing literature strongly suggests that PKCε modulates the binge drinking trait by mediating the development of acute functional tolerance. However, studies using chronic models suggest that PKCε is not directly involved in chronic ethanol-induced behavioral changes, at least in the rat amygdala. It will be interesting to observe and compare PKCε changes in both acute and chronic ethanol models.

## Author contributions

JK: Conceptual framework and design; MM and JK: Searched references; AH, MM, EY, NA, FJ, RP, and JK: Drafted manuscript; JK: Preparation of figure; AH, MM, EY, NA, FJ, RP, and JK: Critically revised the manuscript.

### Conflict of interest statement

The authors declare that the research was conducted in the absence of any commercial or financial relationships that could be construed as a potential conflict of interest.
